# Iterative Neighbour-Information Gathering for Ranking Nodes in Complex Networks

**DOI:** 10.1038/srep41321

**Published:** 2017-01-24

**Authors:** Shuang Xu, Pei Wang, Jinhu Lü

**Affiliations:** 1 School of Mathematics and Statistics, Xi’an Jiaotong University, Xi’an 710049, China; 2 School of Mathematics and Statistics, Henan University, Kaifeng 475004, China; 3 Laboratory of Data Analysis Technology, Henan University, Kaifeng 475004, China; 4 Institute of Systems Science, Academy of Mathematics and Systems Science, Chinese Academy of Sciences, Beijing 100190, China

## Abstract

Designing node influence ranking algorithms can provide insights into network dynamics, functions and structures. Increasingly evidences reveal that node’s spreading ability largely depends on its neighbours. We introduce an iterative neighbourinformation gathering (Ing) process with three parameters, including a transformation matrix, a priori information and an iteration time. The Ing process iteratively combines priori information from neighbours via the transformation matrix, and iteratively assigns an Ing score to each node to evaluate its influence. The algorithm appropriates for any types of networks, and includes some traditional centralities as special cases, such as degree, semi-local, LeaderRank. The Ing process converges in strongly connected networks with speed relying on the first two largest eigenvalues of the transformation matrix. Interestingly, the eigenvector centrality corresponds to a limit case of the algorithm. By comparing with eight renowned centralities, simulations of susceptible-infected-removed (SIR) model on real-world networks reveal that the Ing can offer more exact rankings, even without a priori information. We also observe that an optimal iteration time is always in existence to realize best characterizing of node influence. The proposed algorithms bridge the gaps among some existing measures, and may have potential applications in infectious disease control, designing of optimal information spreading strategies.

Evidences show the heterogeneous connectivity^1^,^2^ of real-world complex networks ranging from biology^3^,^4^,^5^ to socio-tech^6^,^7^,^8^ science, where the understanding of significant role that a single node plays provides insights into network structure and functions^9^,^10^. Ranking or identifying the node importance gains attention of a growing number of researchers from different disciplines^11^,^12^,^13^,^14^,^15^, since it’s the first step to optimize the epidemic^10^ or information diffusion in viral marketing^16^, to more efficiently control systems^11^, to design search engines^17^, to reduce the dimension of networks^18^,^19^, to understand the hierarchical organization of biological networks^4^,^12^, to develop strategies for improving the resilience of transport networks^20^, to prioritize resource allocation for upgrading of hierarchical and distributed networks^21^, as well as to predict the nodes with cohesion of the whole structure in multilayer networks^22^.

Numerous researchers focus on how to rank node importance from epidemic dynamics^10^,^23^,^24^,^25^,^26^,^27^,^28^. Degree, the number of a node’s linkages, is the simplest and intuitive indicator, specially in networks with broad degree distributions^23^. Traditionally, large degree nodes (also called hubs) are deemed as important nodes^24^. While, Kitsak *et al*.^10^ stated that the position of node (measured by coreness), identified by *k*-core decomposition analysis^29^, plays a more critical role in epidemic spreading in four real-world networks. Recently, Chen *et al*.^30^ reported that the clustering hinders propagation in some social networks and proposed a ClusterRank (CR) algorithm with low computational complexity.

Degree, coreness and CR estimate propagation capability of network nodes from different perspectives, which take the impact of linkage quantity, position, and clustering into account, respectively. Recently, many centralities based on neighbour’s information have been proposed, such as semi-local^25^, extended neighbours’ coreness^26^ (ENC), improved neighbours’ *k*-core^27^ (INK) and H-index^28^, providing us with more accurate and reliable ranking results. H-index of a node is defined as the maximum integer *h* such that the considered node has at least *h* neighbours whose degrees are greater than *h*. Higher H-index indicates that the node has a number of neighbours with high degree. Compared with degree, H-index can better capture the spreading importance. A node with higher degree does infect many neighbours at start times, while the spread process will cease quickly if its neighbours have low degrees. Nevertheless, the case may be improved for high H-index node whose neighbours also with lots of neighbours. Therefore, increasingly evidences show that propagation capability largely depends on information from neighbours^25^,^26^,^27^,^28^. Many non-neighbour based centralities barely capture single node information, which is too microcosmic to express the macroscopic attribute (i.e. spread ability). An ideal centrality is better to contain more neighbours’ information and reflect more global structural features of the target network.

Though the mentioned H-index is a pretty paradigm, it only collects information (degree) of one-layer neighbours, which leads to low resolution. In order to more exactly predict node importance and comprehensively capture the node propagation feature, we need a new technique to sufficiently embrace information from more layers of neighbours. Motivated by it, we develop a new general framework to rank nodes through gathering neighbour’s information combined with a priori knowledge iteratively. A new algorithm is introduced, which is called iterative neighbour-information gathering (Ing), the process assigns each node with an Ing score representing node importance. The Ing process has three parameters 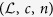, where 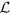 denotes a well defined linear transformation, which can automatically gather neighbour’s information, *c* denotes a priori information or called initial score, and *n* denotes gathering time. It is proved that the iterative algorithm converges when *n* tends to infinity, regardless of initial scores. The steady state is just the eigenvector centrality or cumulative nomination, provided that a special 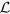 is set. It is noted that all the states in the Ing process can be viewed as different centrality measures. To evaluate whether the Ing score can estimate node importance, we apply the SIR model^31^ on six representative real-world networks. Simulations show that if parameters are properly chosen, the Ing process will obtain more exact rankings, compared with degree, H-index^28^, coreness^10^, closeness^32^, betweenness^33^, LeaderRank (LR)^34^, weighted LeaderRank (WLR)^35^ and CR^30^. Further investigations reveal that the Ing score without a priori information still outperforms these eight traditional centralities.

## Results

### Iterative neighbour-information gathering algorithm

In the following, we propose the new algorithm. Denote *G(V, E*) as a given complex network, where *V* and *E* are the sets of nodes and edges, respectively. |*V*| = *v* represents the number of nodes, and |*E*| = *m* denotes the number of edges. The network can be directed or undirected, weighted or unweighted, connected or unconnected, depends on the edge set *E* or the adjacency matrix *A* = (*a*_*ij*_)_*v*×*v*_. If there is an edge from node *i* to node *j, a*_*ij*_ is non-zero, otherwise, *a*_*ij*_ = 0. If all the non-zero values 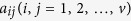 are equal, then the network is unweighted, otherwise, the network is weighted. Moreover, *a*_*ii*_ = 1 indicates a self-loop for node *i*.

#### 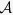 -Ing process

Firstly, for node *i*, we choose a certain centrality *c*_*i*_ as it’s initial Ing score. The initial Ing score of node *i* is taken as the benchmark centrality 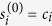, which represents the a priori information. Denote 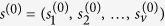
′ as the 0-order Ing score vector. Subsequently, the Ing process relies on a linear transformation 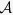 to collect neighbours’ information. Naturally, we define the matrix corresponding to 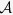 as network’s adjacency matrix *A*, mapping the benchmark centrality space into the Ing space. After the initial setting, we define


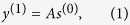



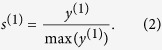


where *s*^(1)^ is called 1-order Ing score vector. *s*^(1)^ is the percentage transformation of *y*^(1)^. Specifically, for node *i*, the 1-order Ing score can be obtained as


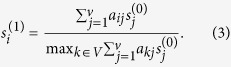


Similarly, we can define *n*-order Ing score vector as


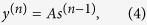



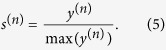


As a matter of fact, the free parameter *n* can be viewed as the collected layers of neighbour-information. Via sprawling on adjacency matrix, the Ing score will collect information of more nodes with the increasing of *n*. In summary, the flows of the Ing algorithm are as follows:

Step 1. Select certain a priori information as an initial Ing score *s*^(0)^;

Step 2. Apply linear transformation 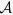 to (*n* − 1)-order Ing score *s*^(*n*−1)^, and obtain 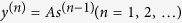;

Step 3. Normalize *y*^(*n*)^ by its maximum component, and derive the *n*-order Ing score vector 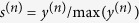.

Since the algorithm is based on 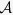, we therefore call the algorithm as the 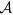-Ing process.

#### 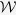 -Ing process

The linear transformation in the Ing process can be freely defined. 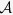-Ing process gathers a priori information of neighbours, but weakens the power of a considered node itself. Therefore, we further define a new transformation 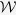, whose corresponding matrix is *W* = *A* + *I*, where *I* is the identity matrix. Mixed information of the node and its neighbours is included in the 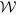-Ing. Generally speaking, the Ing score will be determined if parameters 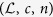 are set, where 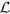 is a linear transformation defined by practical demands, *c* is a benchmark centrality or called a priori information, and *n* is the iteration time. In the following analysis, we mainly focus on 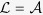 and 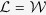.

**Figure 1:**
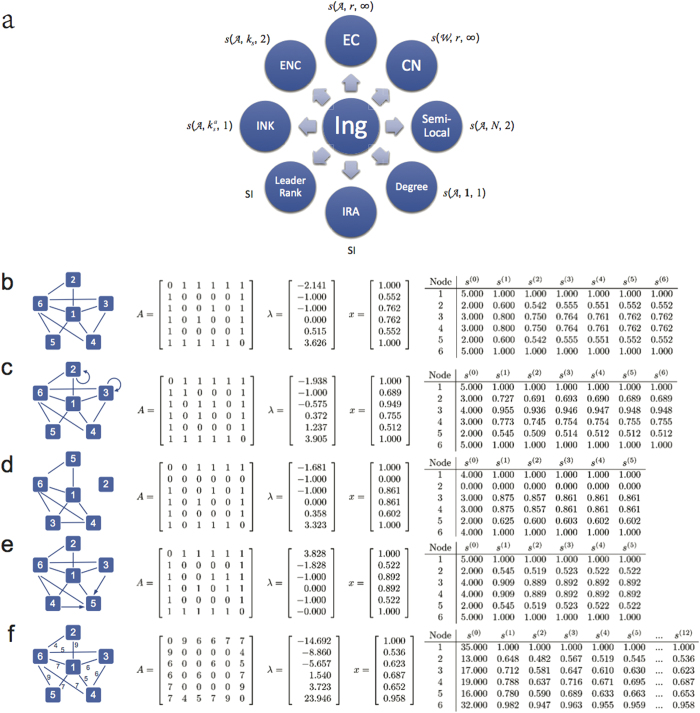
Relationships between the Ing score and some other centralities. (**a**) Relationships of the Ing score with EC, CN, semi-local, degree, IRA, LeaderRank, INK, ENC. Here, EC, CN and IRA denote eigenvector centrality, cumulative nomination and iterative resource allocation. *r* denotes an arbitrary vector, *k*_*s*_ denotes the coreness, *a* is a tunable parameter, **1** denotes an all-one vector, *N* denotes the number of the first nearest neighbours and the second ones. The parameter settings for the LeaderRank and the IRA are a little complex, for more information, see Supporting Information. (**b**) A toy example for an undirected, connected and unweighted network. (**c**) A toy example for an unweighted, undirected and connected network with loops. (**d**) A toy example for an unweighted, undirected and unconnected network without loop. (**e**) A toy example for an unweighted, connected and directed network. Bidirectional edges are shown without arrows. (**f**) A toy example for a connected, undirected and weighted network. In each example, *A* represents adjacency matrix, *λ* denotes the eigenvalues of *A, x* denotes the corresponding dominate eigenvector.

**Figure 2:**
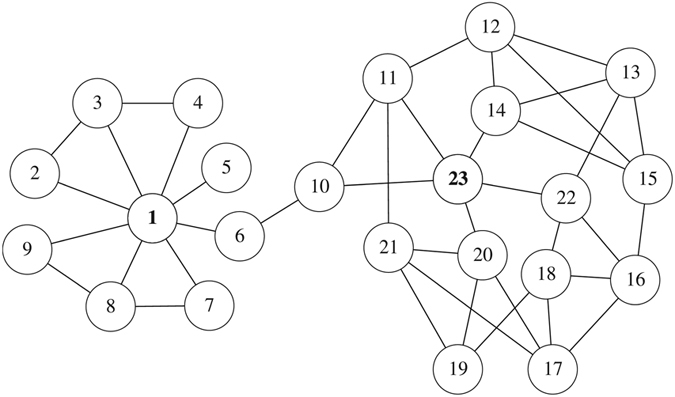
A toy network with 23 nodes25.

The proposed Ing process can bridge the gaps among many existing measures. Figure 1(a) gives the relationships of the Ing with the other measures. The Ing score includes the eigenvector centrality, cumulative nomination, the semi-local centrality, the degree, IRA, LeaderRank, INK, ENC as its special cases. For example, 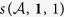 corresponds to the degree centrality, where **1** denotes the vector whose elements are all ones; 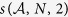 corresponds to the semi-local centrality, where *N* denotes the number of the first nearest neighbours and the second ones; 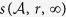 corresponds to the eigenvector centrality, where *r* is any kind of a priori information. 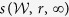 corresponds to the cumulative nomination. From this point of view, the eigenvector centrality and the cumulative nomination stand for the global collected information, while low-order Ing score stands for the local one. To see the equivalence of the Ing score with the other measures, we also consider several toy examples, as shown in Fig. 1(b–d), where *c* = degree and 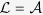. We consider five different types of networks, including directed or undirected, connected or unconnected, weighted or unweighted, with or without self-loops. From Fig. 1(b–d), on the one hand, it demonstrates that the proposed algorithm can be used in any types of networks. On the other hand, it shows that all the Ing scores of the five toy networks converge to their eigenvector centralities for several rounds of iterations. Moreover, to intuitively verify the equivalence between the Ing process and the degree, semi-local centrality, we consider another toy example with 23 nodes^25^, the toy network is shown in Fig. 2, the degree and the semi-local centrality for the network are shown in Table 1. For the toy example, if we set the initial centrality as an all-one vector 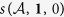, subsequently after one step of iteration, we get 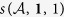, which is equivalent to the degree (equal after perform percentage transformation to the degree vector). If we set the initial centrality as 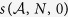, subsequently after two steps of iterations, 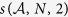 is equivalent to the semi-local centrality.

For more information about the algorithm and its applications in small networks, one can refer to Supplementary Information. From the toy examples as shown in Fig. 1, we conclude that the Ing score is prone to achieve a steady state with the increasing of iterations. In fact, we can obtain the following theorem to support the convergence of the Ing process.

**Theorem 1** *For any type of complex network G(V, E*), *set linear transformation* 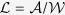 *whose matrix is L, the Ing score vector sequence s*^(*n*)^ *converges. Specially, provided that a complex network is strongly connected, the limit state of the Ing process corresponds to the dominant eigenvector of L. The convergence speed of the algorithm depends on the ratio of the largest eigenvalue of L to the second largest one*.

The proof of Theorem 1 is based on the Perron-Frobenius theorem and ref. 36. For details, see the Methods section. In the following sections, we will show the performance of the new algorithms, and compare it with some traditional measures. More importantly, we illustrate that long iteration time of the Ing process does not always benefit for ranking influence. The best result with optimal n* will be obtained in low-order Ing space.

### Quantifying spreading influence

Spread dynamics is the most common process in many domains, such as physics, biology and society. In order to evaluate the effectiveness of the Ing process on quantifying spreading influence, we employ the SIR model^31^ to simulate the spreading process, where the influence of node *i* is denoted by spread range *R*_*i*_, computed by the average number of recovered and infected nodes at the steady states of the SIR process after 1000 independent simulations, and each simulation begins with node *i* as the single infection seed (see more on SIR model in Methods). We apply the Kendall *τ (τ*_*b*_) correlation coefficient^37^ to quantify prediction accuracy, where this non-parameter measurement can well abstract the correlation. *τ* lies in [−1, 1], greater absolute value of *τ* implies higher correlation between two sample vectors (see more on Kendall *τ* in Methods). Higher correlation between the Ing score vector and the spread range vector indicates better prediction accuracy of the Ing process. Six representative real-world networks are considered. The basic statistical measurements are shown in Table 2. The Email network^38^ is a communication network, the Jazz^39^ and NS^40^ are collaboration networks, the PB^41^ is a information network, the Router^42^ is a technological network, the USAir^43^ is a transportation network (see more on dataset description in Methods). The sizes of the six networks range from 198 to 5022, with average degrees range from 2.49 to 27.70. Except the Router network, all the other networks are with very high clustering coefficients. The Email and the Jazz are assortative, while the other four networks are all disassortative. To evaluate the performance of the new algorithm, eight widely used traditional centralities are considered to be a priori information, including the degree, H-index^28^, coreness^10^, closeness^32^, betweenness^33^, LR^34^, WLR^35^ and CR^30^, all of which are representative and express different structure attributes of the target network (see more on centrality definitions in Methods).

The Kendall *τ* correlation coefficients between different centralities and spreading ranges are shown in Tables 3, 4 and 5. Table 3 is for ordinary centralities and Tables 4 and 5 are for 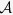
- and 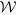-Ing score with optimal iteration time n*. On one hand, it shows that the optimal Ing score always significantly outperforms the ordinary centralities. The greatest improvement is 39.88% (for NS, the best Ing score is set as parameter (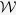, closeness) with *τ* = 0.7983, the best ordinary centrality is the WLR with *τ* = 0.5707). The lowest improvement is 0.95% (for PB, the best Ing score is set as (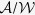, H-index) with *τ* = 0.8401, the best ordinary centrality is the H-index with *τ* = 0.8321). On the other hand, the upper bound of ordinary centralities’ *τ* is inferior to the lower bound from the Ing score. That is, regardless of what kind of a priori information is chosen, even the least relevant Ing score can give more accurate result. Tables 4 and 5 also suggest that the 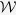-Ing process will gain higher optimal correlations than the 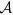-Ing process. Because information from a node itself is included in the 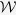-Ing process, which increases its distinguish ability.

**Figure 3:**
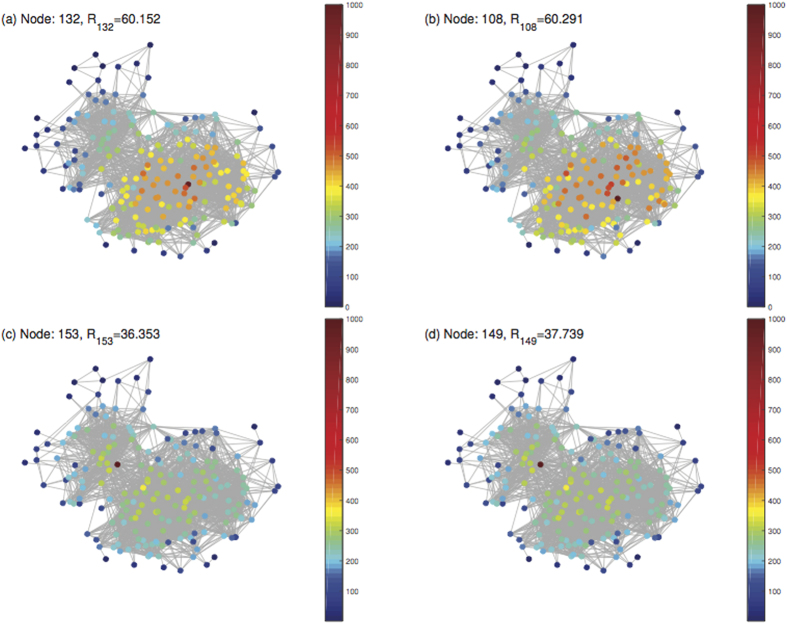
Spread ranges of the Jazz network under four single spreaders. Node’s infection frequency relies on its colour, i.e. blue, green, red mean low, middle, high frequency, respectively. (**a**) Node 132 as an initial spreader, which is a top-5 ranked node according to the *s*(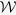, betweenness, 4). (**b**) Node 108 as an initial spreader, which is a top-5 ranked node according to the *s*(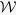, betweenness, 4). (**c**) Node 153 as an initial spreader, which is a top-5 ranked node according to the betweenness. (**d**) Node 149 as an initial spreader, which is a top-5 ranked node according to the betweenness.

To further show the superiority of the Ing process, the Jazz network will be explored in detail. The top-5 nodes ranked by the betweenness and the *s*(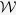, betweenness, 4) are {136, 153, 60, 149, 168} and {60, 136, 132, 168, 108}, respectively. The different nodes in the two lists, namely, 108, 132, 149 and 153, are considered. We choose these nodes as a single propagation seed successively and run 1000 independent simulations for each case. Frequency that a node is recovered or infected at a stable state of the spreading process are counted, we draw the related states of the networks, as shown in Fig. 3. It is obvious that the seed nodes have frequency = 1000. If more nodes have higher frequencies, this seed is supposed to be more influential. We can observe that nodes 132 or 108 as initial spreaders can averagely infect more nodes than that with nodes 153 or 149 as initial spreaders, which indicates the Ing process can offer more exact rankings than the traditional betweenness centrality.

**Figure 4:**
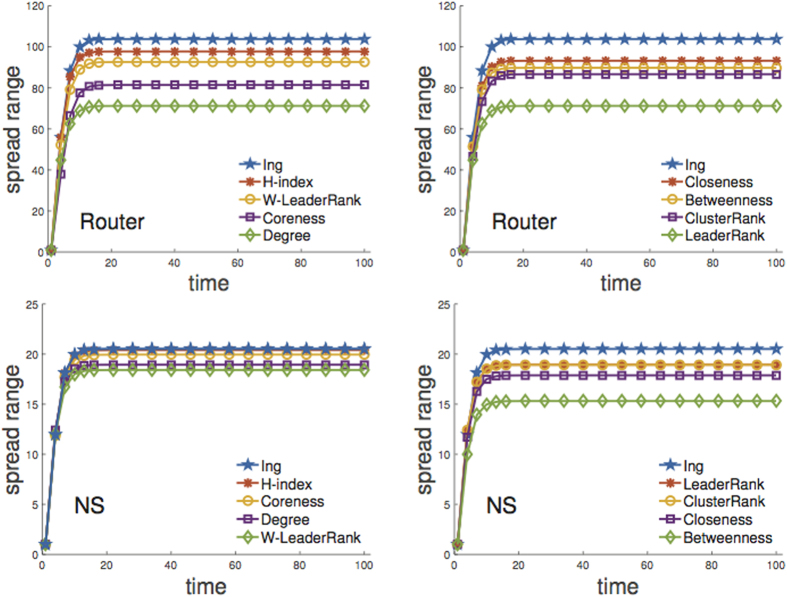
Evolutions of spread ranges for the top-ranked nodes in the Router and the NS networks. The curves are averaged over the top-5 and the top-10 ranked nodes, respectively. For the NS, the node lists identified by the LeaderRank and the ClusterRank are the same, therefore, the two curves coincide with each other. The same figures with error bars can be referred to Supplementary Fig. S2.

In practice, people tend to only concern super spreaders. Now we further show that nodes with higher Ing score do spread wider. The Router and the NS are took as representative examples, and we draw the evolution curves of spread ranges with time. Here we select top-ranked nodes as a single infection seed successively, then average *R*_*i*_ over the top-ranked list. For example, the top-5 node list identified by the degree is {1, 2, 3, 4, 5}. We set node 1 as infection seed, apply the SIR model and then obtain the spread range time series 
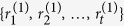
. The series for node 2, 3, 4, 5 are obtained similarly. At last, the spread range time series for degree is averaged over these 5 nodes. Figure 4 shows the average evolution curves for the Router and the NS over top-5 and top-10 ranked nodes. The parameters are chosen as (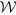, H-index, 4) for the Router, and (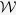, closeness, 5) are chosen for the NS network. Figure 4 reveals that the Ing scores always have the highest average steady spread range in the two networks under the two cases, and indicates the proposed Ing algorithm outperforms the other centralities on spreading ranges.

**Figure 5:**
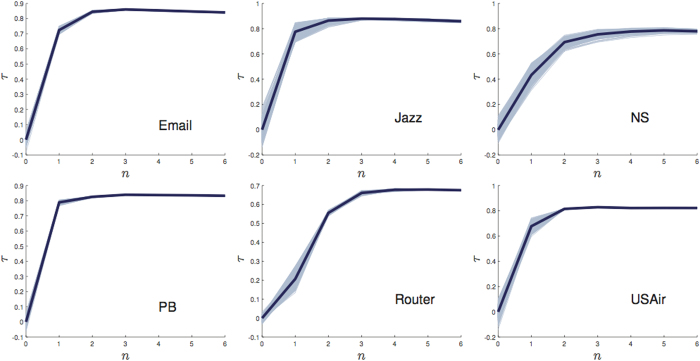
Evolutions of correlation coefficient between spreading range and 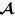
**-Ing score with the increasing of iteration time for the six networks.** The bold lines correspond to the average results over 1000 independent simulation runs.

In large-scale networks, though topology is known, some kinds of a priori information is not easy to be obtained, such as the betweenness and the coreness. Does the prediction accuracy of the Ing process largely depend on its a priori information? Without proper a priori information, does the Ing process still offer exact ranking results? Now we apply the 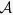-Ing process without a priori information to estimate the spread ability of nodes. A random vector is set as the initial benchmark centrality, whose elements are sampled from uniform distribution between 0 and 1. Figure 5 shows the average correlation between spreading ranges and the 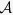-Ing score with random initial centrality, where 1000 random vectors are created, and we define


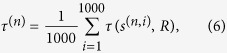


where *s*^(*n,i*)^ is the *n*-order Ing score vector with the *i*’th random vector, and *R* is the spreading range vector. The *τ*^(0)^ is around zero, meaning that the random initial centralities provide nothing information for the prediction. However, the correlation coefficient is improved significantly with *n* increasing from 0 to 6, and the correlation coefficient curves tend to increase with the increasing of the iteration step *n*. The 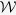-Ing process has similar performance (see Supporting Information). The second columns of Tables 4 and 5 correspond to the *τ* for the optimal Ing score without a priori information. Our results indicate that the Ing score without a priori information is even superior to some tradition measures with a priori information, such as the coreness, the CR, the betweenness in many networks. Therefore, the Ing score can be robustly applied in large-scale networks, which can provide exact rankings as well.

### Optimal iteration time of the Ing process

**Figure 6:**
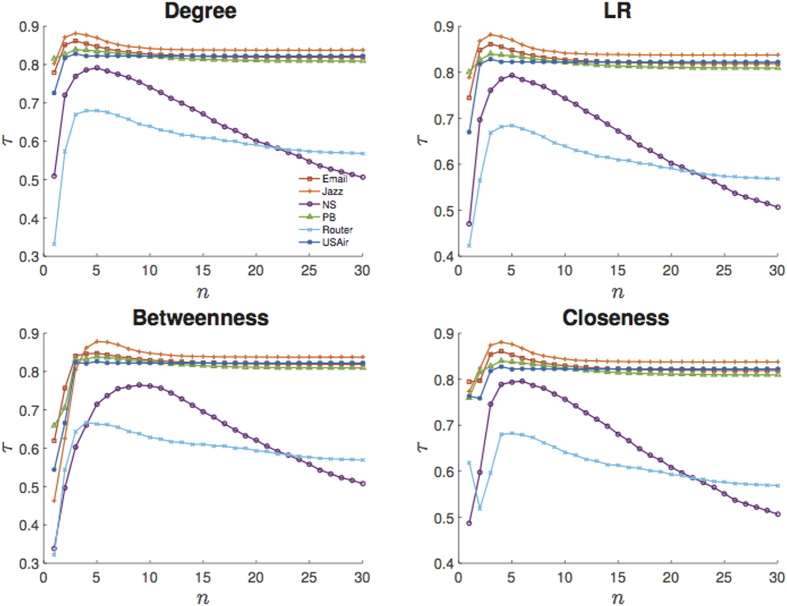
Evolutions of correlation coefficient between spreading range and 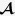-Ing score with four kinds of a priori information. The same figures with error bars can be referred to Supplementary Fig. S3.

Different iteration time *n* corresponds to a different Ing score. It is interesting to explore the effect of *n* on prediction accuracy. Figure 6 shows the evolutions of *τ* with the increasing of iteration time *n*, where only four representative benchmark centralities and the 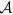-Ing are shown (For 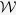-Ing, see Supporting Information). For each case, at the first several iteration steps, *τ* increases linearly with *n*, and quickly reaches a peak value. Then *τ* slowly decreases and eventually it tends to converge to a stable state when *n* is sufficiently large, which further supports our assertion in theorem 1. Figure 6 indicates that we can always obtain the best prediction accuracy if we properly set the iteration time *n*. In fact, from Fig. 6, the best prediction results for the six networks can be obtained when *n* is low. The greatest *τ* and the corresponding optimal n* are shown in Tables 4 and 5 for the six real-world networks. Obviously n* depends on three factors: the benchmark initial centrality, network topology and linear transformation of the Ing process.

The n* for the closeness and the betweenness tends to be larger than the other a priori information. These two centralities are related to the shortest path length, while the other measures are based on node degree. It is noticed that since degree reflects the number of node’s neighbours, accordingly, a priori information of these centralities contains more neighbour knowledge than the closeness and the betweenness. When using the closeness and the betweenness as initial iteration vectors, more time are needed to obtain more neighbour knowledge. We observe that n* for the NS and the Router tend to be larger than the other networks, which may result from their low wiring density. The NS and the Router are sparser and with intensely low average degree, which may hinder information spreading, so more neighbour-information needs to be collected in order to get more prediction accuracy. In seldom cases, the 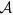-Ing tends to obtain optimal correlation more quickly than the 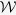-Ing process, while it is not true for most situations. Hence the linear transformation can weakly affect n*.

In conclusion, non-neighbour based benchmark initial centralities, sparser network topology all can affect the optimal iteration time n*. The optimal n* will be larger if the connection density of the target network is smaller and a priori information is based on non-neighbour centralities.

### Discussions and Conclusions

Node ranking, or influential node identification for complex networks is still an open issue. From the viewpoint of statistics and machine learning, this task is a kind of unsupervised learning, i.e. learning process without a guider. We assume that importance of a node largely relies on its neighbours. To collect neighbour information automatically and predict exact ranking list, we propose a new iterative algorithm, called the iterative neighbour-information gathering (Ing) process. The Ing process assigns a score 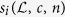 to node *i*, where the three parameters represent linear transformation, a priori information (benchmark centrality), iteration time, respectively. For node *i*, when *n*→∞, the Ing score converges to the *i*’th element of principal eigenvector that corresponds to the matrix of linear transformation 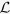, provided that the network is strongly connected. Two proper transformations, 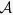 and 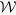 are introduced in this paper. Specially, a limit case of the 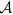-Ing score is the eigenvector centrality. Many existing centralities can be viewed as the special cases of the Ing score. Additionally, except the 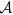 and 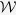 transformations, more general transformations can be defined, such as one can define 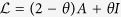, where 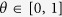 is a weighted parameter. *θ* = 0 is equivalent to the 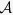-Ing, *θ* = 1 corresponds to the 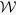-Ing process. One can freely tune *θ* to assign weight on neighbour’s information *A* and self information *I*.

The Ing process can be deemed as a Bayesian style algorithm. The Ing needs *a priori belief*, which may be obtained by our knowledge of nodes or some other centralities, such as degree, coreness, or even pure surmising. Then we apply the properly defined transformation 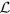 to correct existing belief, i.e. *s*^(1)^ = *Ls*^(0)^, where 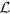 captures the node-to-node connection diagram. 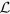 corresponds to a transition matrix *L* and *s*^(*k*)^ denotes *posterior probability* after *k* steps of corrections. The correction steps give us a new knowledge of node importance. We can update it repeatedly by *s*^(*n*)^ = *Ls*^(*n*−1)^. Experimental results show that the update time does not always benefit node ranking, but fortunately the Ing process has a *self-defence mechanism*– the convergence of *s*^(*n*)^, to prevent a low-accuracy result. Moreover, an optimal iteration time is always in existence to realize best characterizing of node influence, and the optimal iteration time depends on the initial centralities and the network topology.

All the indices with different linear transformation, a priori information and iteration time are centralities that can characterize each node’s importance. We demonstrate that the Ing process enhances nodes ranking exactness very much, even without a priori information. Actually, the Ing score will be more accurate when a priori information is included. If *n* is properly set, the Ing score always outperforms many other centralities. In practice, the best prediction result will be obtained when *n* is small. The optimal n* will be delayed if the connection density of the target network is smaller and a priori information is based on non-neighbour centralities.

The Ing process is with computational complexity *O(v* + *m*), where *v* and *m* represent the numbers of the nodes and edges, respectively. Compared with the other global centralities, such as the closeness with *O(vm* + *v*^2^ log *v*), the betweenness with *O(vm* + *v*^2^ log *v*) or *O(vm*), the eccentricity with *O(v*^3^)^44^, the Ing process is rather computationally simple.

Though we mainly consider six undirected networks. the framework of the proposed Ing process is appropriate for all types of complex networks. We discuss the Ing algorithm for directed networks in the Supporting Information. To check the performance in directed networks, we choose out-degree, out-Hindex, out-coreness, LR, WLR, CR as a priori information and take them to compare with our algorithm. Based on the SIR model and six representative directed networks, we illustrate that the Ing score still gives exact ranking performance in directed networks.

At last, we discuss the comparison between our algorithm and the PageRank. The PageRank is one of the best known ranking algorithm^17^, which mimics the behaviour of a net surfer, i.e. one would randomly open a link on current web page, and at the same time will turn to other web pages with a small probability. Even though the PageRank has been applied to various fields, it is reported that the PageRank may be not suitable for disease dynamic^34^. Indeed, compared with the Ing process, the PageRank can not offer better prediction (See details in Supplementary Information). We suspect that there are two reasons for the consequence. On one hand, though the PageRank and the Ing process are both iterative algorithms, the former requires steady score (i.e. *t* = ∞) and the latter often select immediate score (i.e. iteration time *t* is finite). We have demonstrated that larger *t* does not always benefit prediction (see Fig. 6). On the other hand, it is improper to apply the PageRank to describe disease propagation. In the PageRank, a node may receive score out of thin air from a randomly selected website with a small probability, which makes no sense in disease dynamic. While for the Ing process based on the SIR model, a node can be affected with a probability only and only if there are infected neighbours for the node. Thus, we conclude that the Ing is different from the PageRank, and the proposed algorithm has its advantages.

The proposed algorithm bridges the gaps among many existing measures, and includes the eigenvector centrality as a limit case. The proposed algorithm may have potential applications in infectious disease control, designing of optimal information spreading strategies.

## Methods

### Proof of Theorem 1

To prove Theorem 1, we introduce the following lemmas.

**Lemma 1** Ref. 36 *Suppose* 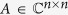 *and u*^(0)^ *is an arbitrary column vector whose components are not all zeros. Let the sequences v*^(*s*)^ *and u*^(*s*)^ *be defined by equations*


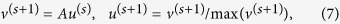


*where the notation* max(*x) denotes the element of maximum modulus of the vector x. Clearly, we have*


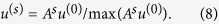


*If eigenvalues of A satisfy* 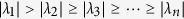, *we have*


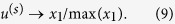


*That is, this sequence* {*u*^(*s*)^} *converges to eigenvector corresponding to the dominant eigenvalue λ*_1_. *The convergence speed depends on* |*λ*_1_|/|*λ*_2_|. *Faster convergence will be obtained if* |*λ*_1_|/|*λ*_2_| *is larger. If there are a number of independent eigenvectors corresponding to the dominant eigenvalue λ*_1_, *this does not affect the convergence. Actually, if*  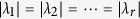 *and* 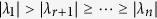, *we have*


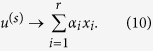


*So in this case, the iterations tend to some vector lying in the subspace spanned by the eigenvectors x*_1_, *x*_2_, …, *x*_*r*_, *and the limit depends upon the initial vector u*^(0)^.

**Lemma 2** (**Perron**-**Frobenius Theorem**) Refs 45, 46 *Let A be an irreducible non-negative n* × *n matrix with spectral radius ρ(A*) = *r. Then the following statements hold. 1) The number r is a positive real number and it is an eigenvalue of the matrix A, called the Perron-Frobenius eigenvalue. 2) The Perron-Frobenius eigenvalue r is simple and its eigenspace is one-dimensional. 3) A has an eigenvector x with eigenvalue r whose components are all positive*.

For the case of a complex network *G(V, E*) is strongly connected, if we can associate a matrix *L* with a certain directed graph *G*_*L*_, it has exactly *n* nodes, where *n* is size of *L*, and there is an edge from node *i* to node *j* precisely when *L*_*ij*_ > 0. Then, the matrix *L* is irreducible if and only if its associated graph *G*_*L*_ is strongly connected. Since *G(V, E*) is strongly connected, matrix *A* and *W* can be associated a strongly connected graph, that is, they are irreducible. According to Lemma 2, we have that the eigenspace of the dominant eigenvalue of *A* or *W* is one-dimensional. Hence, using Lemma 1, the Ing score vector sequences *s*^(*n*)^ converge to the dominant eigenvector.

For the case of a complex network *G(V, E*) is not strongly connected, the dimension of the eigenspace of the dominant eigenvalue of *A* or *W* may be more than one. Of course, the Ing score vector sequences *s*^(*n*)^ still converge, but the limit states are not unique and rely on its initial states.

### Traditional Node Centralities

*Degree centrality* is the most simplest indicator, defined as the number of neighbours of a node. *Semi-local centrality*^25^ of node *b* is defined as


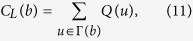



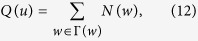


where *N(w*) is the number of the nearest and the second nearest neighbours of node *w*, 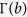 is the neighbour set of node *b*. Chen *et al*. argued that local clustering tends to play the negative role in spreading process, then proposed the *ClusterRank* algorithm^30^, defined as


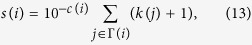


where *c(i*) is the local clustering coefficient of node *i* and *k(j*) is the degree of node *j*. Lü *et al*. applied *H-index*^28^ to complex network. H-index of a node is *h*, the maximum integer such that there are at least *h* neighbours, each of which have degree greater than *h*.

Kitsak *et al*.^10^ argued that, especially in network with broader degree distribution, the *coreness k*_*s*_ can well identify spreading power of a node. The coreness is a score assigned by k-core decomposition analysis. At first, we remove nodes with degree *k* = 1 and this may cause new nodes with *k* ≤ 1 who are supposed to be removed until remained ones with degree *k* ≥ 2. Nodes removed in this step consist of 1-layer. Then we remove nodes with *k* = 2 and new nodes with *k* ≤ 2 until remained ones with *k* ≥ 3, and those nodes consist of 2-layer. The process continues until all nodes are removed and a node coreness is the number of layers where it locates at. The *neighbourhood coreness* and *extended neighbourhood coreness*^26^ are defined respectively as


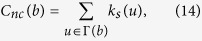



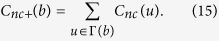


The *improved neighbour*’*s k-core*^27^ is defined as


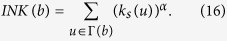


Measures in the LR-family^34^,^35^,^47^ are based on the random diffusion process. Given a network with *v* nodes and *m* edges, the LR algorithm adds a ground node that connects with all others via bidirectional edges resulting in a strongly connected network, then applies the standard random walk process in order to assigns a score to each node. Initially, scores are given as *s*_*g*_(0) = 0 for ground node and *s*_*i*_(0) = 1 for ordinary nodes; then scores are updated by the rule


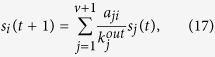


where *a*_*ji*_ is 1 if node *j* point to node *i* and 0 otherwise, 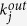 is the out-degree of node *j*. It’s shown that the process will converge soon and stable score *s*(∞) is used to evaluate node’s spreading ability. This method outperforms the well-known PageRank for prediction accuracy and robustness against noisy data. Li *et al*.^35^ proposed *weighted-LeaderRank* whose update rule follows


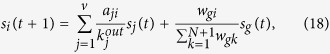


where 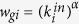 and *α* is a tunable parameter. Without loss of generality, we set *α* = 1.

*Betweenness centrality*^33^ of node *i* measures the fraction of the shortest paths passing through it, defined by


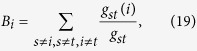


where *g*_*st*_ is the number of the shortest paths between node *s* and *t*, and *g*_*st*_(*i*) is the number of the shortest paths between node *s* and *t* that pass through node *i. Closeness centrality*^32^ of node *i* measures how far from *i* to all other nodes. In this paper, it is defined as


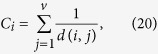


where *d(i, j*) is the length of the shortest path between *i* and *j*.

### SIR model

The SIR model, referring to susceptible-infected-recovered model, is widely used in epidemics and information spreading. In the SIR model, there are three states for all nodes. An infected node will recover with probability *α* and its neighbours will be infected with probability *β*. In the simulations, we set *α* = 1 and *β* = 1.5*β*_*c*_, where 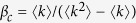 is the approximation of epidemic threshold. Epidemic strength is defined as *β*/*α*, if the epidemic strength was higher than the epidemic threshold *β*_*c*_, then the information or disease can be spread out, while the infected numbers will be exponential decreased if *β*/*α* < *β*_*c*_^48^,^49^,^50^. The chosen *β* and *α* guarantee that *β*/*α* > *β*_*c*_, and information can be spread out on the networks. To eliminate the fluctuation of *R*_*i*_ in different simulation runs, we average our results over 1000 independent simulation runs.

### Kendall correlation coefficient

Kendall *τ*_*b*_ correlation coefficient^37^ is a popular rank correlation statistical measure. Considering *n* samples of two variables 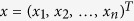 and 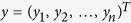, paired samples (*x*_*i*_, *y*_*i*_) and (*x*_*j*_, *y*_*j*_) are concordant if (*x*_*i*_ − *x*_*j*_) (*y*_*i*_ − *y*_*j*_) > 0, discordant if (*x*_*i*_ − *x*_*j*_) (*y*_*i*_ − *y*_*j*_) < 0, or they are neither concordant nor discordant if (*x*_*i*_ − *x*_*j*_) (*y*_*i*_ − *y*_*j*_) = 0. In fact, if (*x*_*i*_ − *x*_*j*_) (*y*_*i*_ − *y*_*j*_) = 0, one can deduce that *x*_*i*_ = *x*_*j*_ or *y*_*i*_ = *y*_*j*_, and we call *x*_*i*_ = *x*_*j*_ and *y*_*i*_ = *y*_*j*_ as ties of *x* and *y*, respectively. There are totally *n(n* − 1)/2 pairs of samples. Based on the number of concordant and discordant pairs, then the Kendall *τ*_*b*_ correlation coefficient is defined as


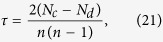


where *N*_*c*_ and *N*_*d*_ are the numbers of concordant and discordant pairs, respectively.

### Datasets

1. Email^38^: The email communication network at the University Rovira i Virgili in Tarragona in the south of Catalonia in Spain. Nodes are users and each edge represents that at least one email was sent. The direction of emails or the number of emails are not stored.
2. Jazz^39^: The collaboration network between Jazz musicians. Each node is a Jazz musician and an edge denotes that two musicians have played together in a band. The data was collected in 2003.
3. NS^40^: A coauthorship network of scientists working on network theory and experiment, as compiled by Newman in May 2006. Node is a scientist and edge represent that two scientist wrote at least one joint work. The original network have 1589 nodes, and only the largest connected component is considered.
4. PB^41^: A network contains front-page hyperlinks between blogs in the context of the 2004 US election. A node represents a blog and an edge represents a hyperlink between two blogs. The original network is directed, here is its undirected version.
5. Router^42^: A network of autonomous systems of the Internet connected with each other. Nodes are autonomous systems (AS), and edges denote communication.
6. USAir^43^: The US air transportation network. Nodes are airports, edges represent airways.

## Additional Information

**How to cite this article**: Xu, S. *et al*. Iterative Neighbour-Information Gathering for Ranking Nodes in Complex Networks. *Sci. Rep.* **7**, 41321; doi: 10.1038/srep41321 (2017).

**Publisher's note:** Springer Nature remains neutral with regard to jurisdictional claims in published maps and institutional affiliations.

## Supplementary Material

Supplementary Information

## Figures and Tables

**Table 1:** The 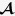 -Ing scores for a toy network with 23 nodes in Fig. 2.

Node	*k*	Scaled *k*	SL	Scaled SL	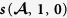	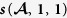	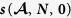	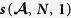	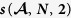
1	8	1.000	145	0.725	1	1.000	9	1.000	0.725
2	2	0.250	92	0.460	1	0.250	8	0.254	0.460
3	3	0.375	101	0.505	1	0.375	8	0.373	0.505
4	2	0.250	92	0.460	1	0.250	8	0.254	0.460
5	1	0.125	67	0.335	1	0.125	8	0.134	0.335
6	2	0.250	104	0.520	1	0.250	11	0.269	0.520
7	2	0.250	92	0.460	1	0.250	8	0.254	0.460
8	3	0.375	101	0.505	1	0.375	8	0.373	0.505
9	2	0.250	92	0.460	1	0.250	8	0.254	0.460
10	3	0.375	111	0.555	1	0.375	9	0.552	0.555
11	4	0.500	166	0.830	1	0.500	12	0.612	0.830
12	4	0.500	157	0.785	1	0.500	9	0.567	0.785
13	4	0.500	157	0.785	1	0.500	8	0.582	0.785
14	4	0.500	166	0.830	1	0.500	9	0.597	0.830
15	4	0.500	156	0.780	1	0.500	9	0.552	0.780
16	4	0.500	158	0.790	1	0.500	11	0.582	0.790
17	4	0.500	158	0.790	1	0.500	9	0.582	0.790
18	4	0.500	148	0.740	1	0.500	9	0.597	0.740
19	3	0.375	119	0.595	1	0.375	8	0.418	0.595
20	4	0.500	158	0.790	1	0.500	10	0.597	0.790
21	4	0.500	148	0.740	1	0.500	9	0.582	0.740
22	4	0.500	170	0.850	1	0.500	12	0.627	0.850
23	5	0.625	200	1.000	1	0.625	14	0.776	1.000

*k* denotes degree and SL denotes semi-local. The scaled ones are divided by their maximum value.

**Table 2:** Topological features of six real-world networks.

Network	*v*	*m*	〈*k*〉	*C*	*r*
Email	1133	5451	9.62	0.254	0.078
Jazz	198	2742	27.70	0.633	0.020
NS	379	914	4.82	0.798	−0.082
PB	1222	16714	27.36	0.360	−0.221
Router	5022	6258	2.49	0.033	−0.138
USAir	332	2126	12.81	0.749	−0.208

*v* and *m* are numbers of nodes and links. 〈*k*〉 denotes the average degree. *C* and *r* represent the clustering^51^ and assortative coefficients^47^,^52^, respectively.

**Table 3:** Kendall *τ* correlation coefficients between spreading range and traditional centralities.

Network	Degree	H-index	Coreness	Closeness	Betweenness	LR	WLR	CR
Email	0.7794	**0**.**8103**	0.8021	0.7747	0.6195	0.7443	0.7959	0.7347
Jazz	0.8021	**0**.**8431**	0.7958	0.6961	0.4629	0.7885	0.8216	0.7550
NS	0.5092	0.5178	0.4747	0.3510	0.3392	0.4710	**0**.**5707**	0.4644
PB	0.8159	**0**.**8321**	0.8274	0.7375	0.6589	0.8002	0.8124	0.7591
Router	0.3309	0.2877	0.2946	**0**.**5975**	0.3228	0.4222	0.4549	0.5675
USAir	0.7256	0.7540	0.7529	0.7453	0.5442	0.6695	**0**.**7717**	0.6061

**Table 4:** Kendall *τ* correlation coefficients between spreading range and the 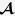 -Ing score with optimal iteration time n*.

Network	Random	Degree	H-index	Coreness	CR	LR	WLR	Betweenness	Closeness
Email	0.8603 (3)	**0**.**8615** (2)	0.8613 (2)	0.8595 (2)	0.8513 (2)	**0**.**8615** (2)	0.8610 (2)	0.8478 (4)	0.8608 (3)
Jazz	0.8795 (3)	0.8816 (2)	**0**.**8826** (2)	0.8758 (2)	0.8719 (2)	0.8814 (2)	0.8816 (2)	0.8778 (4)	0.8803 (3)
NS	0.7861 (6)	0.7910 (4)	0.7775 (4)	0.7681 (5)	0.7884 (4)	0.7932 (4)	0.7832 (3)	0.7646 (8)	**0**.**7957** (5)
PB	0.8392 (3)	0.8394 (2)	**0**.**8395** (1)	0.8378 (3)	0.8379 (2)	0.8394 (2)	**0**.**8395** (2)	0.8385 (4)	0.8394 (3)
Router	0.6787 (6)	0.6796 (4)	**0**.**6897** (4)	0.6860 (3)	0.6818 (3)	0.6843 (4)	0.6839 (3)	0.6677 (3)	0.6821 (4)
USAir	0.8284 (3)	0.8284 (2)	0.8376 (1)	**0**.**8398** (1)	0.8303 (2)	0.8285 (2)	0.8274 (2)	0.8260 (4)	0.8273 (3)

The integers in parentheses is the optimal n* corresponding to the greatest *τ*. Nine kinds of a priori information are considered.

**Table 5:** Kendall *τ* correlation coefficients between spreading range and the 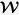 -Ing score with optimal iteration time n*.

Network	Random	Degree	H-index	Coreness	CR	LR	WLR	Betweenness	Closeness
Email	0.8602 (3)	0.8615 (2)	**0**.**8621** (2)	0.8608 (3)	0.8510 (2)	0.8612 (2)	0.8617 (2)	0.8481 (4)	0.8613 (3)
Jazz	0.8795 (3)	0.8818 (2)	**0**.**8829** (2)	0.8757 (2)	0.8713 (2)	0.8820 (2)	0.8821 (2)	0.8770 (4)	0.8806 (3)
NS	0.7861 (6)	0.7933 (4)	0.7793 (4)	0.7740 (5)	0.7897 (4)	0.7925 (4)	0.7869 (4)	0.7643 (10)	**0**.**7983** (5)
PB	0.8391 (3)	0.8394 (2)	0.8394 (1)	0.8381 (3)	0.8381 (2)	0.8394 (2)	**0**.**8395** (2)	0.8386 (4)	0.8393 (3)
Router	0.6854 (5)	0.6869 (4)	**0**.**6934** (4)	0.6888 (4)	0.6881 (2)	0.6870 (4)	0.6883 (4)	0.6703 (3)	0.6868 (4)
USAir	0.8276 (3)	0.8285 (2)	**0**.**8372** (1)	0.8363 (1)	0.8291 (2)	0.8285 (2)	0.8272 (2)	0.8250 (4)	0.8274 (3)

The integers in parentheses is the optimal n* corresponding to the greatest *τ*. Nine kinds of a priori information are considered.
