# Supplementary material for: Iterative Neighbour-Information Gathering for Ranking Nodes in Complex Networks
Source: Sci Rep. 2017 Jan 24;7:41321. doi: 10.1038/srep41321 (PMC5259765; doi:10.1038/srep41321)
Supplement: Supplementary Information [file srep41321-s1.pdf]

Supplementary Information for Iterative Neighbour-Information Gathering for Ranking Nodes in Complex Networks

Shuang Xu[1,2], Pei Wang[2,3,*], and Jinhu Lü[4]

[1]School of Mathematics and Statistics, Xi'an Jiaotong University , Xi'an 710049, China
[2]School of Mathematics and Statistics, Henan University, Kaifeng 475004, China
[3]Laboratory of Data Analysis Technology, Henan University, Kaifeng 475004, China
[4]Institute of Systems Science, Academy of Mathematics and Systems Science, Chinese Academy of Sciences, Beijing 100190, China
[*]wp0307@126.com,wangpei@henu.edu.cn

Examples of the Ing process in toy networks

To understand the Ing process, we apply it to some toy networks, as shown in Fig. S1. To illustrate how to calculate the Ing scores, for the first toy network as shown in the first panel of Fig. S1, we set $\mathscr{L} = \mathscr{A}$ and $c = Degree$. We can use matrix format to obtain all nodes' Ing score at one time as follows:

$$y^{(1)} = As^{(0)} = \begin{bmatrix} 0 & 1 & 1 & 1 & 1 & 1 \\ 1 & 0 & 0 & 0 & 0 & 1 \\ 1 & 0 & 0 & 1 & 0 & 1 \\ 1 & 0 & 1 & 0 & 0 & 1 \\ 1 & 0 & 0 & 0 & 0 & 1 \\ 1 & 1 & 1 & 1 & 1 & 0 \end{bmatrix} \begin{bmatrix} 5 \\ 2 \\ 3 \\ 3 \\ 2 \\ 5 \end{bmatrix} = \begin{bmatrix} 15 \\ 10 \\ 13 \\ 13 \\ 10 \\ 15 \end{bmatrix}, \tag{1}$$

$$s^{(1)} = \frac{y^{(1)}}{\max(y^{(1)})} = \begin{bmatrix} 1 \\ 0.6 \\ 0.8 \\ 0.8 \\ 0.6 \\ 1 \end{bmatrix}. \tag{2}$$

If the network size is too large to be expressed as a matrix, we prefer the following format:

$$y_1^{(1)} = \sum_{i=1}^{6} a_{1i} s_i^{(0)} = \sum_{i \in \Gamma(1)} s^{(0)} = 2+3+3+2+5 = 15, \tag{3}$$

$$y_2^{(1)} = \sum_{i=1}^{6} a_{2i} s_i^{(0)} = \sum_{i \in \Gamma(2)} s^{(0)} = 5+5 = 10, \tag{4}$$

$$y_3^{(1)} = \sum_{i=1}^{6} a_{3i} s_i^{(0)} = \sum_{i \in \Gamma(3)} s^{(0)} = 5+3+5 = 13, \tag{5}$$

$$y_4^{(1)} = \sum_{i=1}^{6} a_{4i} s_i^{(0)} = \sum_{i \in \Gamma(4)} s^{(0)} = 5+3+5 = 13, \tag{6}$$

$$y_5^{(1)} = \sum_{i=1}^{6} a_{5i} s_i^{(0)} = \sum_{i \in \Gamma(5)} s^{(0)} = 5+5 = 10, \tag{7}$$

$$y_6^{(1)} = \sum_{i=1}^{6} a_{6i} s_i^{(0)} = \sum_{i \in \Gamma(6)} s^{(0)} = 5+2+3+3+2 = 15, \tag{8}$$

where $\Gamma(i)$ is the neighbour set of node i. The maximum is $\max(y^{(1)}) = 15$, then we obtain the 1-order Ing score $s^{(1)} = y^{(1)}/15$. Similarly, higher-order Ing score can be obtained using either of the two calculation methods. The evolution of the Ing score for the network are shown in the second figure of Fig. S1, the detailed calculating process are omitted. It's noticed that the Ing score vector converges when $n = 5$. For the other networks, we find that self-loops, disconnection, direction and weight can not damage the convergence of the process.

Fig. 4 and Fig. 6 with error bars

Curves in Fig. 4 and Fig. 6 are averaged over 1000 independent simulation runs. The plots with error bars are shown in Fig. S2 and Fig. S3. It is reported that the conclusions still hold, even though we take the error bars into account.

Relationship of the Ing score and the traditional centralities

Some existing centralities can be viewed as special cases of the Ing process, such as the degree, semi-local centrality, the eigenvector centrality, the LeaderRank and the iterative resource allocation (IRA). The equivalence between the Ing process and the degree, semi-local centrality, the eigenvector centrality are discussed in the main text. For the LeaderRank and the IRA, the settings of these two algorithms are a little complicated. Now we first introduce the work flows of the LeaderRank and the IRA.

Given a complex network $G(V,E)$, V and E are node and edge sets respectively, $|V| = v$ and $|E| = m$ denotes the number of nodes and edges respectively. Its adjacency matrix is $A = (a_{ij})$, where $a_{ij} = 1$ if node i points to node j and 0 otherwise. First of all, the LeaderRank adds a new node, which connects with all nodes via bidirectional edges, to make the network strongly connected. The new node is called ground node and others are called ordinary nodes. The new network's adjacency matrix is

$$\widetilde{A} = \begin{bmatrix} A & \mathbf{1} \\ \mathbf{1}' & 0 \end{bmatrix}, \tag{9}$$

where $\mathbf{1}$ is a $n \times 1$ vector whose elements are all 1. Then, the LeaderRank assigns a score to each node, $s_g^{(0)} = 0$ for ground node and $s_i^{(0)} = 1$ for ordinary nodes. The LeaderRank score is updated by the rule:

$$s_i^{(n)} = \sum_{j=1}^{v+1} \frac{a_{ji}}{k_j^{out}} s_j^{(n-1)}. \tag{10}$$

We can define the matrix of the Ing process as $Z = (z_{ij})$, where $z_{ij} = \frac{a_{ji}}{k_j^{out}}$. Hence, the LeaderRank is equivalent to $s(\mathscr{Z}, r, \infty)$, where r is a random vector whose elements are not all zeros. Remark that, in fact, the initial Ing score is chosen randomly and it does not affect the limit state, while the original LeaderRank is set as $(\mathbf{1}', 0)'$. The network is augmented via ground node, so the Ing score vector is $(v+1)$-dimensional, while we only focus on the first v elements.

a

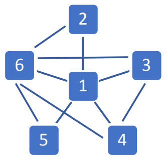

$$A = \begin{bmatrix} 0 & 1 & 1 & 1 & 1 & 1 \\ 1 & 0 & 0 & 0 & 0 & 1 \\ 1 & 0 & 0 & 1 & 0 & 1 \\ 1 & 0 & 1 & 0 & 0 & 1 \\ 1 & 0 & 0 & 0 & 0 & 1 \\ 1 & 1 & 1 & 1 & 1 & 0 \end{bmatrix} \quad \lambda = \begin{bmatrix} -2.141 \\ -1.000 \\ -1.000 \\ 0.000 \\ 0.515 \\ 3.626 \end{bmatrix} \quad x = \begin{bmatrix} 1.000 \\ 0.552 \\ 0.762 \\ 0.762 \\ 0.552 \\ 1.000 \end{bmatrix}$$

Node	$s^{(0)}$	$s^{(1)}$	$s^{(2)}$	$s^{(3)}$	$s^{(4)}$	$s^{(5)}$	$s^{(6)}$
1	5.000	1.000	1.000	1.000	1.000	1.000	1.000
2	2.000	0.600	0.542	0.555	0.551	0.552	0.552
3	3.000	0.800	0.750	0.764	0.761	0.762	0.762
4	3.000	0.800	0.750	0.764	0.761	0.762	0.762
5	2.000	0.600	0.542	0.555	0.551	0.552	0.552
6	5.000	1.000	1.000	1.000	1.000	1.000	1.000

b

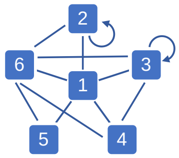

$$A = \begin{bmatrix} 0 & 1 & 1 & 1 & 1 & 1 \\ 1 & 1 & 0 & 0 & 0 & 1 \\ 1 & 0 & 1 & 1 & 0 & 1 \\ 1 & 0 & 1 & 0 & 0 & 1 \\ 1 & 0 & 0 & 0 & 0 & 1 \\ 1 & 1 & 1 & 1 & 1 & 0 \end{bmatrix} \quad \lambda = \begin{bmatrix} -1.938 \\ -1.000 \\ -0.575 \\ 0.372 \\ 1.237 \\ 3.905 \end{bmatrix} \quad x = \begin{bmatrix} 1.000 \\ 0.689 \\ 0.949 \\ 0.755 \\ 0.512 \\ 1.000 \end{bmatrix}$$

Node	$s^{(0)}$	$s^{(1)}$	$s^{(2)}$	$s^{(3)}$	$s^{(4)}$	$s^{(5)}$	$s^{(6)}$
1	5.000	1.000	1.000	1.000	1.000	1.000	1.000
2	3.000	0.727	0.691	0.693	0.690	0.689	0.689
3	4.000	0.955	0.936	0.946	0.947	0.948	0.948
4	3.000	0.773	0.745	0.754	0.754	0.755	0.755
5	2.000	0.545	0.509	0.514	0.512	0.512	0.512
6	5.000	1.000	1.000	1.000	1.000	1.000	1.000

c

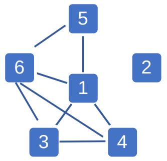

$$A = \begin{bmatrix} 0 & 0 & 1 & 1 & 1 & 1 \\ 0 & 0 & 0 & 0 & 0 & 0 \\ 1 & 0 & 0 & 1 & 0 & 1 \\ 1 & 0 & 1 & 0 & 0 & 1 \\ 1 & 0 & 0 & 0 & 0 & 1 \\ 1 & 0 & 1 & 1 & 1 & 0 \end{bmatrix} \quad \lambda = \begin{bmatrix} -1.681 \\ -1.000 \\ -1.000 \\ 0.000 \\ 0.358 \\ 3.323 \end{bmatrix} \quad x = \begin{bmatrix} 1.000 \\ 0.000 \\ 0.861 \\ 0.861 \\ 0.602 \\ 1.000 \end{bmatrix}$$

Node	$s^{(0)}$	$s^{(1)}$	$s^{(2)}$	$s^{(3)}$	$s^{(4)}$	$s^{(5)}$
1	4.000	1.000	1.000	1.000	1.000	1.000
2	0.000	0.000	0.000	0.000	0.000	0.000
3	3.000	0.875	0.857	0.861	0.861	0.861
4	3.000	0.875	0.857	0.861	0.861	0.861
5	2.000	0.625	0.600	0.603	0.602	0.602
6	4.000	1.000	1.000	1.000	1.000	1.000

d

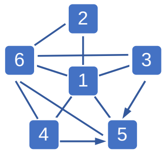

$$A = \begin{bmatrix} 0 & 1 & 1 & 1 & 1 & 1 \\ 1 & 0 & 0 & 0 & 0 & 1 \\ 1 & 0 & 0 & 1 & 1 & 1 \\ 1 & 0 & 1 & 0 & 1 & 1 \\ 1 & 0 & 0 & 0 & 0 & 1 \\ 1 & 1 & 1 & 1 & 1 & 0 \end{bmatrix} \quad \lambda = \begin{bmatrix} 3.828 \\ -1.828 \\ -1.000 \\ 0.000 \\ -1.000 \\ -0.000 \end{bmatrix} \quad x = \begin{bmatrix} 1.000 \\ 0.522 \\ 0.892 \\ 0.892 \\ 0.522 \\ 1.000 \end{bmatrix}$$

Node	$s^{(0)}$	$s^{(1)}$	$s^{(2)}$	$s^{(3)}$	$s^{(4)}$	$s^{(5)}$
1	5.000	1.000	1.000	1.000	1.000	1.000
2	2.000	0.545	0.519	0.523	0.522	0.522
3	4.000	0.909	0.889	0.892	0.892	0.892
4	4.000	0.909	0.889	0.892	0.892	0.892
5	2.000	0.545	0.519	0.523	0.522	0.522
6	5.000	1.000	1.000	1.000	1.000	1.000

e

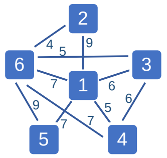

$$A = \begin{bmatrix} 0 & 9 & 6 & 6 & 7 & 7 \\ 9 & 0 & 0 & 0 & 0 & 4 \\ 6 & 0 & 0 & 6 & 0 & 5 \\ 6 & 0 & 6 & 0 & 0 & 7 \\ 7 & 0 & 0 & 0 & 0 & 9 \\ 7 & 4 & 5 & 7 & 9 & 0 \end{bmatrix} \quad \lambda = \begin{bmatrix} -14.692 \\ -8.860 \\ -5.657 \\ 1.540 \\ 3.723 \\ 23.946 \end{bmatrix} \quad x = \begin{bmatrix} 1.000 \\ 0.536 \\ 0.623 \\ 0.687 \\ 0.652 \\ 0.958 \end{bmatrix}$$

Node	$s^{(0)}$	$s^{(1)}$	$s^{(2)}$	$s^{(3)}$	$s^{(4)}$	$s^{(5)}$	...	$s^{(12)}$
1	35.000	1.000	1.000	1.000	1.000	1.000	...	1.000
2	13.000	0.648	0.482	0.567	0.519	0.545	...	0.536
3	17.000	0.712	0.581	0.647	0.610	0.630	...	0.623
4	19.000	0.788	0.637	0.716	0.671	0.695	...	0.687
5	16.000	0.780	0.590	0.689	0.633	0.663	...	0.653
6	32.000	0.982	0.947	0.963	0.955	0.959	...	0.958

Figure S1: 5 toy networks.

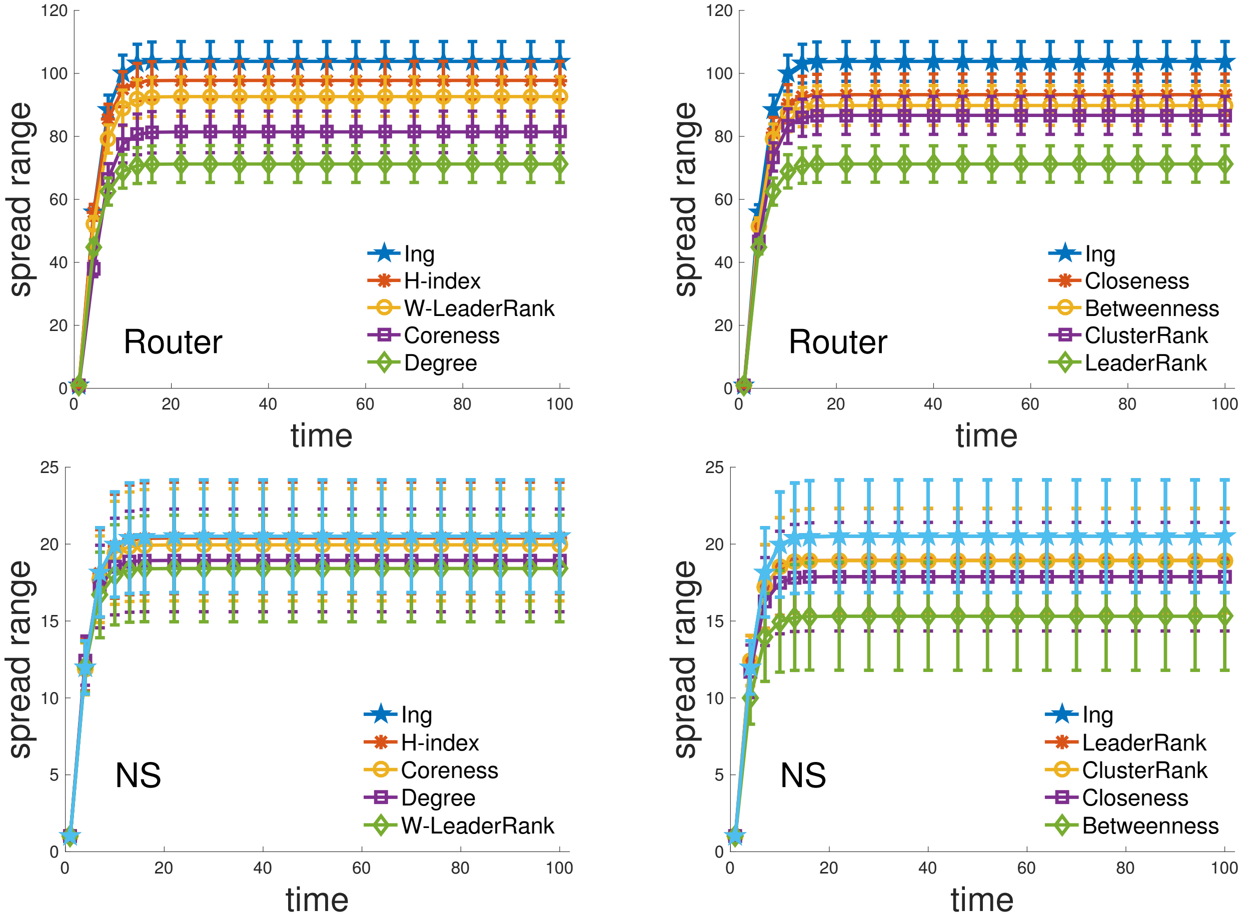

Figure S2: Evolutions of spread ranges for the top-ranked nodes in the Router and the NS networks. The curves are averaged over the top-5 and the top-10 ranked nodes, respectively. For the NS, the node lists identified by the LeaderRank and the ClusterRank are the same, therefore, the two curves coincide with each other.

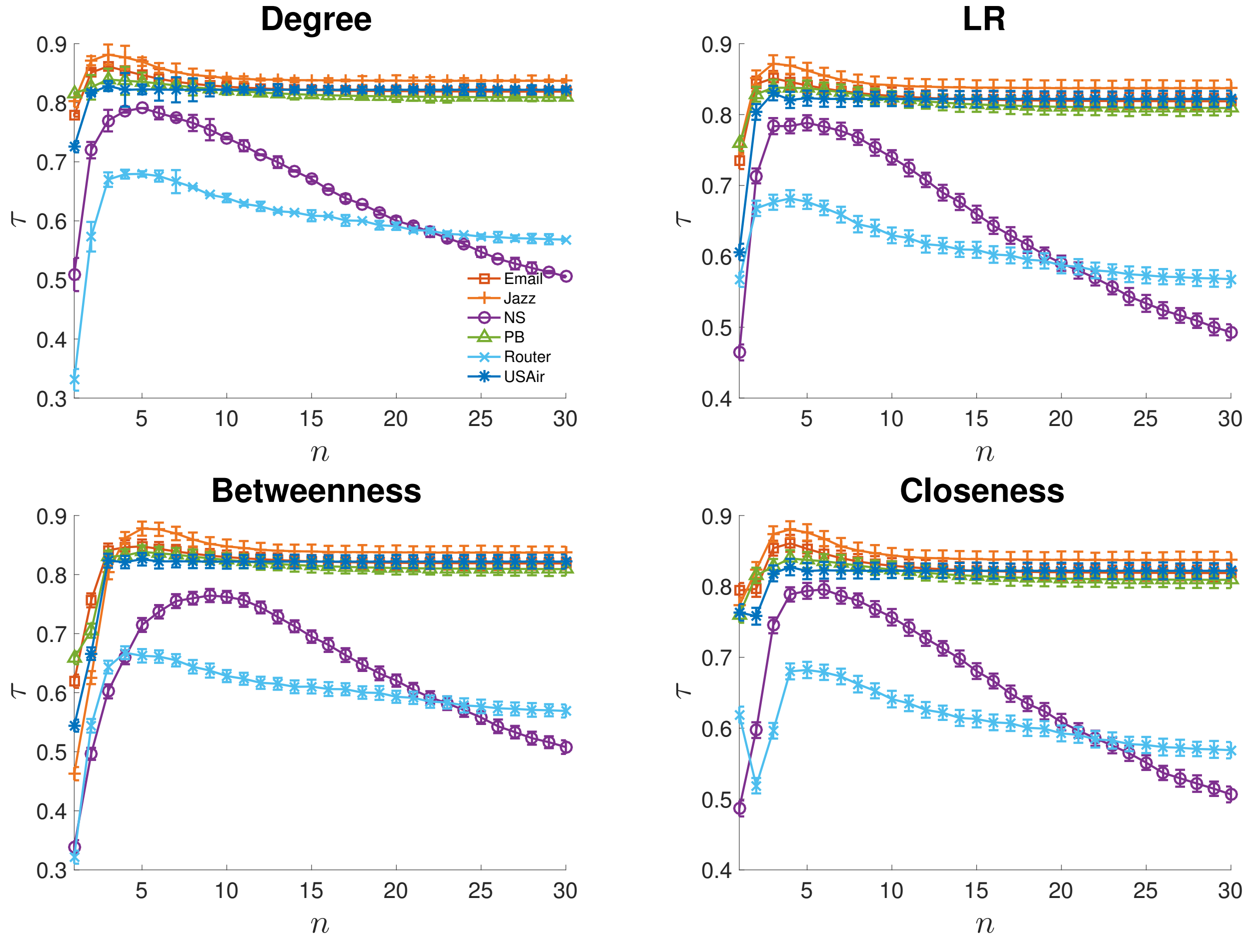

Figure S3: Evolutions of correlation coefficient between spreading range and $\mathscr{A}$-Ing score with four kinds of a priori information.

The IRA also iteratively assigns score to each node, while the update rule is different from the LeaderRank. Its initial scores are set as 1 and its linear transformation matrix is $X = (x_{ij})$, where

$$x_{ij} = \frac{\theta_i^\alpha}{\sum_{u\in\Gamma(j)} \theta_u^\alpha} a_{ij}. \tag{11}$$

θ_i is the prior information of node i, often defined as degree, coreness, closeness, betweenness and so on. Hence, the IRA is equivalent to $s(\mathscr{X}, r, \infty)$.

The $\mathscr{W}$-Ing process

When priori information are absent, the algorithm still works well. We choose linear transformation as $\mathscr{W}$ and priori information as random vector. From Fig. S4, we find that the $\mathscr{W}$-Ing also can improve accuracy remarkably from $n = 0$ to 6 and obtain a pretty result with n^*. To see the evolution of the $\mathscr{W}$-Ing score, we select four kinds of representative priori information and draw Fig. S5. From Fig. S5, we can draw the following conclusions. τ first increases and then decrease, there always a peak value for τ. The peak value corresponds to the optimal iteration times. Moreover, τ tends to be stable when n is sufficiently large.

The Ing process for directed networks

Definition of the Ing process on directed networks

The original Ing algorithm is designed mainly for undirected networks, while it can be generalized for directed ones. Given $G(V,E)$ as a directed network with adjacency matrix $A = (a_{ij})$, where $a_{ij} = 1$ when i points to j and 0 otherwise. Since edges are directional in directed networks, a node i has two types of neighbour, in-neighbour and out-neighbour. The first type are those who point to i and the second are those who are pointed by i. When disease spread via directional edges, only out-neighbours contribute to node i's spread range, hence, the Ing process simply collects out-neighbours' information. Denote $s^{(n-1)}$ as $(n-1)$-order Ing score vector, we have

$$y^{(n)} = As^{(n-1)}, \tag{12}$$

$$s^{(n)} = \frac{y^{(n)}}{\max(y^{(n)})}, \tag{13}$$

where collection matrix A can be replaced by some other well-defined ones, for example, $W = A + I$. Remark that if we have adjacency matrix $A = (a_{ij})$, where $a_{ij} = 1$ when j points i, Eq.(12) should be changed as $y^{(n)} = A^T s^{(n-1)}$.

Datasets description for some directed networks

To verify the effectiveness of the Ing process on directed networks, we select six representative directed networks.

1. Advogato[1] is an online community platform for developers of free software launched in the year 1999. Nodes are users of Advogato and the directed edges represent trust relationships.
2. Anybeat[2], an online community from a public gathering place where one can interact with people from its neighborhood or across the world.
3. RockLake[3] is the food web of Little Rock Lake, Wisconsin in the United States of America. Nodes in this network are autotrophs, herbivores, carnivores and decomposers; links represent food sources.
4. SpaBook[4] reflects word adjacency relationships of a Spanish book. Nodes in the network are words and an edge denotes that two words occurred one after another in the book. The network is directed, i.e., the edge (u, v) denotes that word u was followed by word v. Since a word can occur twice in a row, the network contains loops.
5. USairport[6] is a directed network of flights between US airports in the year 2010. Each edge represents a connection from one airport to another, and the weight of an edge shows the number of flights on that connection in the given direction.
6. UCsocial[5] contains sent messages between the users of an online community of students from the University of California, Irvine. A node represents a user. A directed edge represents a sent message.

The spread rate of these networks are 0.033, 0.0285, 0.15, 0.05, 0.03, 0.05 respectively.

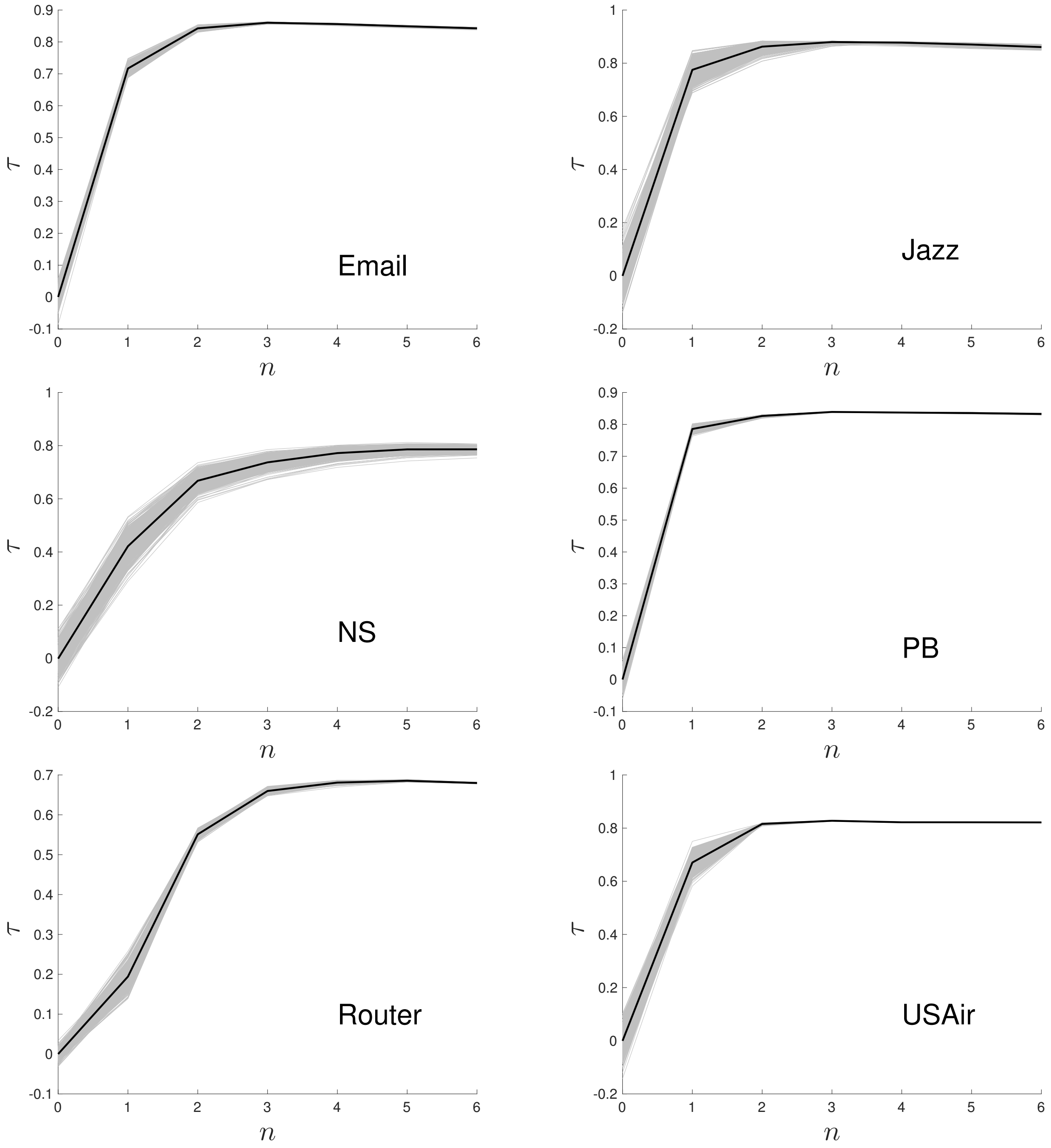

Figure S4: Evolutions of correlation coefficient between spreading range and the $\mathscr{W}$-Ing score with the increasing of iteration times in the six networks. The bold lines in each figure correspond to the average results, which are averaged over 1000 independent simulation runs.

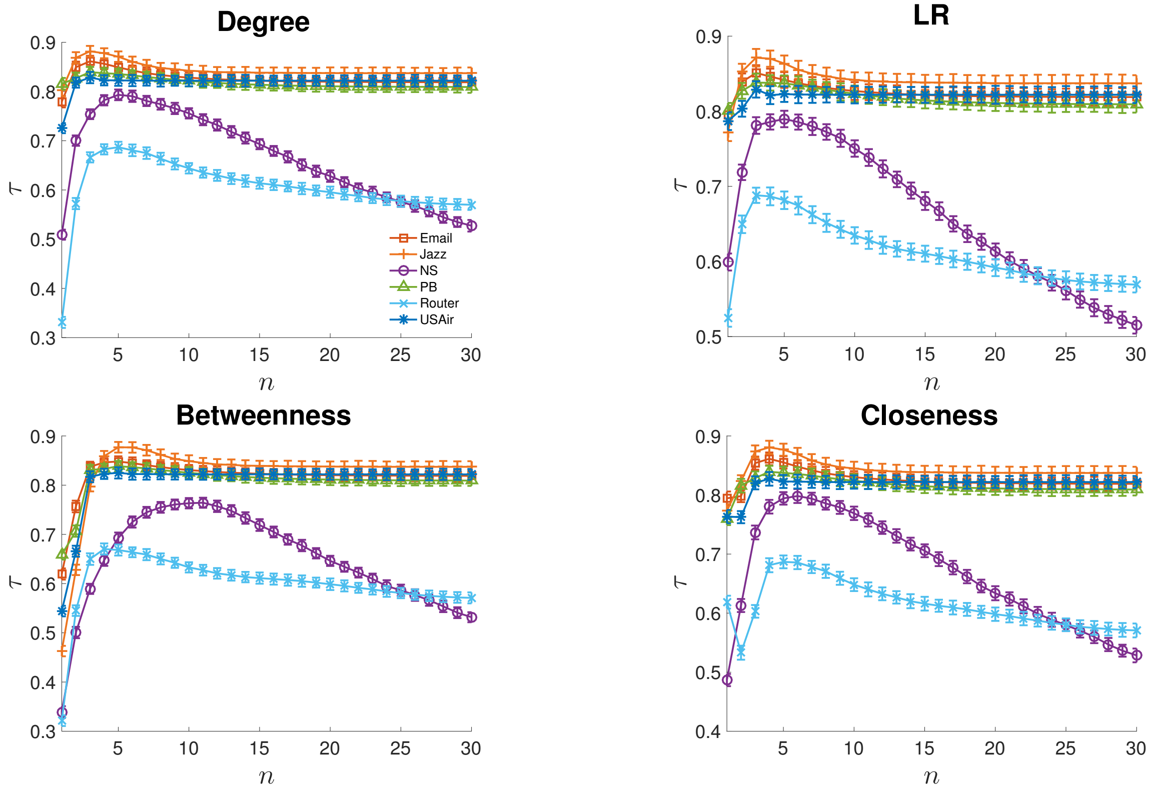

Figure S5: Evolutions of correlation coefficient between spreading range and the $\mathscr{W}$-Ing score under four benchmark centralities with the increasing of iteration times in the six networks.

Table S1: Topological features of six real-world networks. v and m are numbers of nodes and links. $\langle k \rangle$ denote the average degree. C represents the clustering coefficient of connected networks. P_{LCC} and P_{LSCC} are the proportion of the size of largest connected component and largest strongly connected component, respectively.

Network	v	m	$\langle k \rangle$	C	P_{LCC}	P_{LSCC}
Advogato	5042	49631	9.8435	0.5856	1	0.6228
AnyBeat	12645	67053	5.3084	0.3646	1	0.6736
RockLake	183	2494	13.6284	0.3376	1	0.1202
SpaBook	12643	57453	4.5443	0.291	1	0.8686
UCsocial	1899	20296	10.6877	0.0992	0.9968	0.6814
USairport	1574	28236	17.9390	0.6196	0.9987	0.8907

Effectiveness of the Ing process in directed networks

We choose out-degree, out-Hindex, out-coreness, LeaderRank (LR), Weighted LeaderRank (WLR), ClusterRank (CR) as priori information and employ the SIR model to quantify the spreading of node influence. The Kendall τ correlation coefficients between centralities and spread range are shown in Tab. S2. We can also conclude that the Ing score outperforms the others measures.

The difference between Ing process and PageRank

The well-know Google's ranking algorithm, PageRank, has been applied to various issues. PageRank mimics the behavior of a net surfer, i.e. one would randomly open a link on current web page, and at the same time will turn to other web pages with a small probability. In detail, PageRank is iterative just like our Ing process,

$$s_i(t) = q \sum_{j=1}^{N} a_{ji} \frac{s_j(t)}{k_j^{out}} + (1-q)\frac{1}{N}, \tag{14}$$

Table S2: Kendall τ correlation coefficients between centralities and spread range, where k denotes out-degree, h denotes out-Hindex, k_s denotes out-coreness. Each priori information corresponds to three columns, where the first column is the priori information, the second and the third columns are the Ing score at $n = n^*$ with $\mathscr{L} = \mathscr{A}$ and $\mathscr{L} = \mathscr{W}$, respectively. The integers in parentheses is the corresponding optimal n^* with the greatest τ.

Network	k	k-$\mathscr{A}$	k-$\mathscr{W}$	h	h-$\mathscr{A}$	h-$\mathscr{W}$	k_s	k_s -$\mathscr{A}$	k_s -$\mathscr{W}$
advogato	0.8180	0.9236(2)	0.9266(2)	0.8296	0.9203(2)	0.9218(2)	0.8276	0.9204(3)	0.9212(3)
AnyBeat	0.6515	0.9171(2)	0.9191(2)	0.6925	0.9179(1)	0.9157(1)	0.6975	0.9101(3)	0.9088(3)
RockLake	0.7695	0.9044(2)	0.9029(2)	0.8204	0.8991(3)	0.9035(2)	0.7317	0.8858(2)	0.8823(3)
spabook	0.5382	0.571(8)	0.5720(4)	0.5693	0.5770(1)	0.5798(1)	0.5713	0.6658(1)	0.6633(1)
UCsocial	0.9226	0.9602(2)	0.9625(2)	0.9409	0.9791(1)	0.9786(1)	0.9393	0.9617(3)	0.9616(3)
USairport	0.6667	0.9342(2)	0.9336(2)	0.6898	0.9257(2)	0.9251(2)	0.6992	0.9254(2)	0.9248(2)
**Network**	**LR**	**LR-$\mathscr{A}$**	**LR-$\mathscr{W}$**	**WLR**	**WLR-$\mathscr{A}$**	**WLR-$\mathscr{W}$**	**CR**	**CR-$\mathscr{A}$**	**CR-$\mathscr{W}$**
advogato	0.2832	0.9141(4)	0.9082(5)	0.3231	0.9132(5)	0.9077(5)	0.8582	0.9118(2)	0.9138(2)
AnyBeat	0.3903	0.9105(2)	0.8743(2)	0.3922	0.9133(2)	0.8801(2)	0.7720	0.9069(1)	0.9095(1)
RockLake	-0.1629	0.9063(3)	0.9051(4)	-0.1568	0.9063(3)	0.9052(4)	0.8629	0.8956(1)	0.8947(1)
spabook	0.2692	0.5880(2)	0.5585(10)	0.2735	0.5881(2)	0.5585(10)	0.3116	0.5711(5)	0.5709(5)
UCsocial	0.6822	0.9600(3)	0.9214(3)	0.6806	0.9598(3)	0.9193(3)	0.9269	0.9581(3)	0.9603(3)
USairport	0.5700	0.9307(2)	0.9278(2)	0.6495	0.9327(2)	0.9310(2)	0.7078	0.9283(2)	0.9285(2)

where q is a parameter which can be usually set as 0.15. And initially, we set $s(0) = 1$.

Table S3 reports the prediction accuracy of PageRank and $\mathscr{A}$-Ing process. It is shown that Ing process outperforms PageRank in both undirected and directed ones.

Table S3: Kendall τ correlation coefficients between $\mathscr{A}$-Ing / PageRank and spread range. For Ing process family, only the best results are selected.

Network	Email	Jazz	NS	PB	Router	USAir
PageRank	0.6828	0.6941	0.3357	0.7579	0.0591	0.5371
Ing	0.8615	0.8826	0.7957	0.8395	0.6897	0.8398
**Network**	**Advogato**	**Anybeat**	**RockLake**	**SpaBook**	**UCsocial**	**USAirpot**
PageRank	0.6284	0.6483	0.7822	0.5026	0.8945	0.4745
Ing	0.9236	0.9179	0.9063	0.6658	0.9791	0.9342

References

1. Massa, P., Salvetti, M. & Tomasoni, D. Bowling alone and trust decline in social network sites. in: *Proc. Int. Conf. Dependable, Autonomic and Secure Computing*, 658-663 (2009).
2. Fire, M., Puzis, R. & Elovici, Y. Link prediction in highly fractional data sets, in: *Handbook of computational approaches to counterterrorism* (Springer, New York, 2013).
3. Martinez, N. D. Artifacts or attributes? Effects of resolution on the Little Rock Lake food web. *Ecological Monographs* 61, 367-392 (1991).
4. Kunegis, J. *Spanish book network dataset – KONECT*. http://konect.uni-koblenz.de/networks/lasagne-spanishbook (2016) (accessed on Aug. 6, 2016).
5. Opsahl, T. & Panzarasa, P. Clustering in weighted networks. *Social Networks* **31**, 155-163 (2009).
6. Opsahl, T. *Why anchorage is not (that) important: Binary ties and sample selection*. https://toreopsahl.com/2011/08/12/why-anchorage-is-not-that-important-binary-ties-and-sample-selection/ (2011) (accessed on Aug. 6, 2016).
